# Journalistic

**Published:** 1866-03

**Authors:** 


### JOURNALISTIC.

We have before us the first number of the Medical Reporter. a semi-monthly record of medicine and surgery, edited by Drs. J. S. B. Alleyne, and C. F. Potter, St. Louis Mo. The salutatory says, "The pages of the Reporter, will be devoted to original and selected articles, to Correspondence, Domestic and Foreign ; to the Department of Dentistry, and the Collateral sciences, &c."

This is a neatly gotten up Journal, and we feel well assured will be ably conducted. The character of its Editors is such as to preclude any other conclusion. Long may it prosper.

